# Chronic Fluid Flow Is an Environmental Modifier of Renal Epithelial Function

**DOI:** 10.1371/journal.pone.0027058

**Published:** 2011-10-28

**Authors:** Andrew Resnick

**Affiliations:** Department of Physics, Cleveland State University, Cleveland, Ohio, United States of America; University of Houston, United States of America

## Abstract

Although solitary or sensory cilia are present in most cells of the body and their existence has been known since the sixties, very little is been known about their functions. One suspected function is fluid flow sensing- physical bending of cilia produces an influx of Ca^++^, which can then result in a variety of activated signaling pathways. Autosomal Dominant Polycystic Kidney Disease (ADPKD) is a progressive disease, typically appearing in the 5^th^ decade of life and is one of the most common monogenetic inherited human diseases, affecting approximately 600,000 people in the United States. Because ADPKD is a slowly progressing disease, I asked how fluid flow may act, via the primary cilium, to alter epithelial physiology during the course of cell turnover. I performed an experiment to determine under what conditions fluid flow can result in a change of function of renal epithelial tissue. A wildtype epithelial cell line derived the cortical collecting duct of a heterozygous offspring of the Immortomouse (Charles River Laboratory) was selected as our model system. Gentle orbital shaking was used to induce physiologically relevant fluid flow, and periodic measurements of the transepithelial Sodium current were performed. At the conclusion of the experiment, mechanosensitive proteins of interest were visualized by immunostaining. I found that fluid flow, in itself, modifies the transepithelial sodium current, cell proliferation, and the actin cytoskeleton. These results significantly impact the understanding of both the mechanosensation function of primary cilia as well as the understanding of ADPKD disease progression.

## Introduction

Cilia are microtubule-based projections from the cell body (general references http://www.ifcbiol.org/Primaryciliumweb/index.html). Cilia grow from the mature mother centriole of the mother-daughter pair of centrioles in the cell's centrosome [1]. Solitary or sensory cilia are present in most cells of the body and their functions are only now beginning to be defined. Their existence has been known from morphological studies since the sixties [2]; however, because very little has been known about their functions, not much attention has been paid to this particular organelle until recently.

Several observations have illustrated the importance of the solitary cilium: 1) Smell, taste, and vision occurs via modified cilia: olfactory or gustatory sensory cilia and outer segments of rod cell, respectively (see e.g. Figure 15-43 of [3]. 2) Some receptors are specifically located on neurocilia [4]. 3) mutations that occur in proteins resulting in mislocation of normal ciliary proteins or malfunction of solitary cilia itself in the renal epithelium produces a polycystic kidney disease phenotype, which is characterized by the transformation of an absorptive epithelium into a secretory one with formation of cysts [5], [6], [7], [8]. 5) Defects in solitary cilia present during development result in inversion of the normal left-right axis of internal organs, e.g., the heart forms on the right side [9], [10]. Osteocyte cilia have been proposed to be important mechanosensors that determine in part bone remodeling [11]. Additionally, the primary cilium plays a critical role in several essential signaling pathways, including planar cell polarity (PCP) [12], Hedgehog and wnt signaling [13], light and odorant detection [14], and renal mechanosensation [15]. One common motif is the presence of transmembrane receptors and/or ion channels localized to the cilium. A second common motif is the demonstration that physical bending of cilia produces influx of Ca^++^, which can then lead through Ca^++^-induced Ca^++^ release to a Ca^++^ wave spreading through several cells [16], [17], [18].

The microtubule cytoskeleton is organized during cell division to provide the spindle that pulls the chromatides apart into the daughter cells. Under these conditions, cells do not possess cilia. In contrast, when cells enter G_0_ and differentiate, most cells reorganize the microtubule cytoskeleton at one pole of the cell. The mother centrosome becomes the microtubule organizing center from which the single cilium arises. In epithelial cells, such as found in the cortical collecting duct, the cilium projects into the lumen, encased in apical membrane.

Autosomal Dominant Polycystic Kidney Disease (ADPKD) is a progressive disease, typically appearing in the 5^th^ decade of life and is one of the most common monogenetic inherited human diseases, affecting approximately 600,000 people in the United States.

Genetic evidence is quite strong that ciliary dysfunction is an important determinant of renal epithelial cell biology and cystogenesis: 1) The gene products of PKD1 and PKD2 (polycystin 1 and polycystin 2) [8], Tg737 (polaris) [19], and cpk (cystin) [20] localize to the cilium [21] and mutations in these genes result in the transformation of a tubular, absorbing epithelium into a cystic, fluid secreting epithelium with clinical symptoms of polycystic kidney disease [8], [22]. 2) Polycystins 1 and 2 mediate mechanosensation in the primary cilium of kidney cells, native polycystin 2 functions as a plasma membrane Ca^++^-permeable cation channel in renal epithelia [7], [8], and loss of the cilium results in uncontrolled Ca^++^ influx [23]. 3) The deletion of the gene for Kif3ain mature kidneys cells results in renal cyst formation. That is, reprogramming of differentiated renal epithelial cells occurs *after* deletion of the ciliary motor Kif3a and this reprogramming cannot be ascribed to faulty development [6]. 4) Collecting ducts from the orpk/Tg737 mouse possess shortened cilia and develop a cystic phenotype [24], [25]. Taken together, this evidence supports the ‘two-hit’ model of cystogenesis in ADPKD [26]. This model purports to explain the late (5^th^ decade) appearance of cysts as well as the simultaneous appearance of numerous focal cysts through accumulated mutations in mechanosensitive proteins. Abnormalities in ciliary associated proteins result in altered cell functions and disease progression.

Evidence for flow as an environmental factor for ADPKD is currently indirect, and is based mostly on the purported function of the primary cilium [8], [18], putative mechanosensitive proteins (polycystin 1 and polycystin 2 [7], [8], [27], TRPV4 [28], and ENaC [29]), and activated kinase signaling pathways (mTOR [30], STAT6 [27]). Experiments connecting shortened cilia to an ADPKD phenotype have been performed in the presence of fluid flow, illustrating the link between flow sensing and disease state [24], [25]. Experiments have shown phenotypic similarities between constitutively active repair states and the ADPKD state [31], [32], [33].

Because ADPKD is a slowly progressing disease, I asked how fluid flow may act to alter cellular physiology during the course of cell turnover. Cell division in renal epithelia occurs not just during growth or repair of the nephron, but also constitutively. Although not completely understood, it is suspected that renal epithelial cells can de-differentiate, proliferate, and re-differentiate to restore functional integrity of the nephron [34], [35]. Thus, I asked if differentiated epithelial monolayers are sensitive to the flow conditions present during differentiation, and if the flow conditions present during differentiation can alter mechanosensation by the cells after differentiation has occurred. In this way I aim to probe the evolution of renal function in terms of the flow conditions present within a nephron, independent of genetic mutations.

Previously, my laboratory has shown that a cell line derived from the Immortomouse- principal cells of the cortical collecting duct- respond to fluid flow by changing the transepithelial sodium flux, and that this response requires the presence of an intact primary cilium [36]. This cell line is particularly suited for my investigations for two reasons. First, the electrophysiological behavior of this cell type is very simple, consisting of only a few ion channels and transporters. Second, the physiological role of the collecting duct is to ‘fine tune’ the overall salt and water balance in the body (the collecting duct is responsible for the final 0.5% of salt resorption), and so it is logical to probe these cells in the context of slow-acting chronic disease .

We previously demonstrated that ciliary-mediated mechanosensation is very sensitive to flow and that an intact cilium is required to generate a cellular response to flow. Additionally, some of the data suggested that the cellular response involved cellular memory- cells that lost the primary cilium did not attain original levels of transepithelial sodium current after regenerating the cilium.

## Results

### Electrophysiological Results

**Figure 1:**
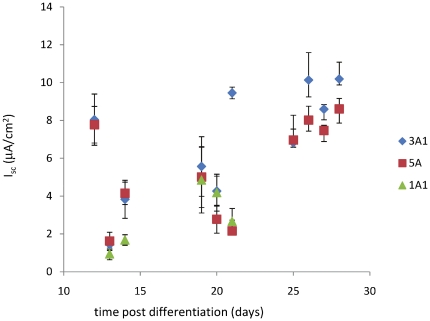
Transepithelial sodium current measurement of monolayers never subjected to fluid flow. The calculated short-circuit equivalent current I_sc_ of each monolayer was measured for several weeks and compared across three different cohorts. It is important to note that the current is due to directed transport of Sodium through the confluent monolayer and reaches a final value approximately 10 days after the confluent monolayers are placed in differentiation conditions. Because I_sc_ is similar (variations are not statistically significant) for monolayers in different experimental groups, the overall experimental approach of comparing the response of different groups of monolayers over time is validated.

The first set of results (Figure 1) compares monolayers from different cohorts never subjected to flow; the presented data shows thatthese monolayers had statistically indistinguishable transepithelial sodium currents, validating our experimental approach.

#### Cells differentiated in the presence of flow exhibit a graded response to flow

**Figure 2:**
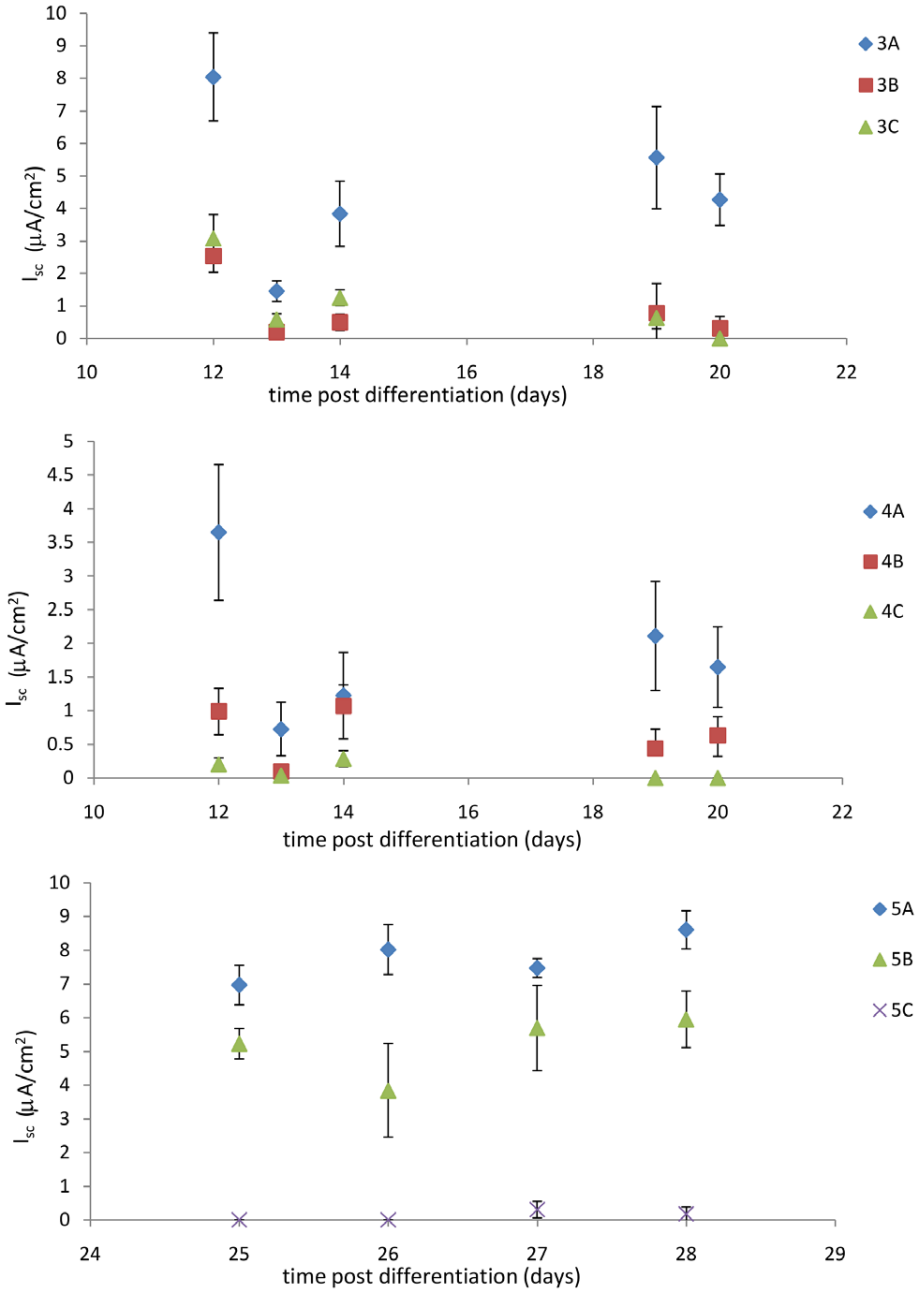
Transepithelial sodium current measurements of Cohort 3 (upper graph) and Cohort 4 (middle graph) and Cohort 5 (lower graph). The calculated short-circuit equivalent current I_sc_ was measured over time and graphed in terms of days after the monolayers were placed in differentiation conditions. All monolayers in Cohort 3 were allowed to differentiate for 10 days in quiescent conditions, and were then maintained in no orbital motion (3A), moved to slow (3B) or rapid (3C) orbital motion at day 10. All monolayers in cohort 4 were exposed to slow orbital motion for 10 days at the onset of differentiation, and were then either removed (4A), maintained at slow motion (4B), or exposed to rapid orbital motion (4C). All monolayers in cohort 5 were held in quiescent differentiation conditions for 20 days and were then either exposed to no (5A), slow (5B), or rapid (5C) orbital motion. The monolayers in Cohort 3 show a response to the presence (points 3B and 3C) or absence (points 3A) of apical fluid flow only, while the monolayers in Cohort 4 and 5 show a graded response to apical fluid flow. Specifically, cells exposed to slow orbital shaking (4B, 5B) had a decreased I_sc_ as compared to monolayers not exposed to orbital motion (4A, 5A) and an increased I_sc_ as compared to monolayers exposed to a more rapid orbital motion (4C, 5C). All variations in I_sc_ are statistically significant as judged by Student's t-test (p<0.01).

Several results obtained previously [17], [25], [36] showed that monolayers respond to the presence or absence of flow only. That is, either the cell responded to flow or it did not- there was no graded response to flow, in contrast to a dose-response curve typical of chemical sensing. Figure 2 provides measurements of cohorts 3, 4 and 5 during the second stage of the shaking protocol, analyzing the response of monolayers to a change in fluid flow conditions. Cohort 3 consisted of cells allowed to differentiate for 10 days under quiescent conditions, while cohort 4 consisted of cells that differentiated under the influence of slow shaking. Cohort 3 did not exhibit a graded response: differentiated cells exposed to fluid flow (3B, 3C) showed a significant decrease in transepithelial sodium current as compared to 3A, but there was no significant difference in response to either slow or fast shaking. By contrast, cohort 4 did show a graded response: cells removed from the orbital shaker (4A) had an increased transepithelial sodium current, while cells exposed to a faster rate of shaking (4C) further reduced the transepithelial sodium current as compared to 4B. This differential response was statistically significant.

Interestingly, monolayers in cohort 5 which differentiated under quiescent conditions for an extended period of time (10 days) before being exposed to flow also exhibited a graded response to changes in flow conditions, similar to cohort 4. Unfortunately, both cohorts 5 and 6 experienced significant contamination during the experiment and are largely excluded from the results.

Examination of the transepithelial sodium flux of Cohorts 1 and 2 are less definitive (data not shown); Cohort 2 appeared to present a graded response to flow while Cohort 1 did not, consistent with the data for cohorts 3 and 4.

**Figure 3:**
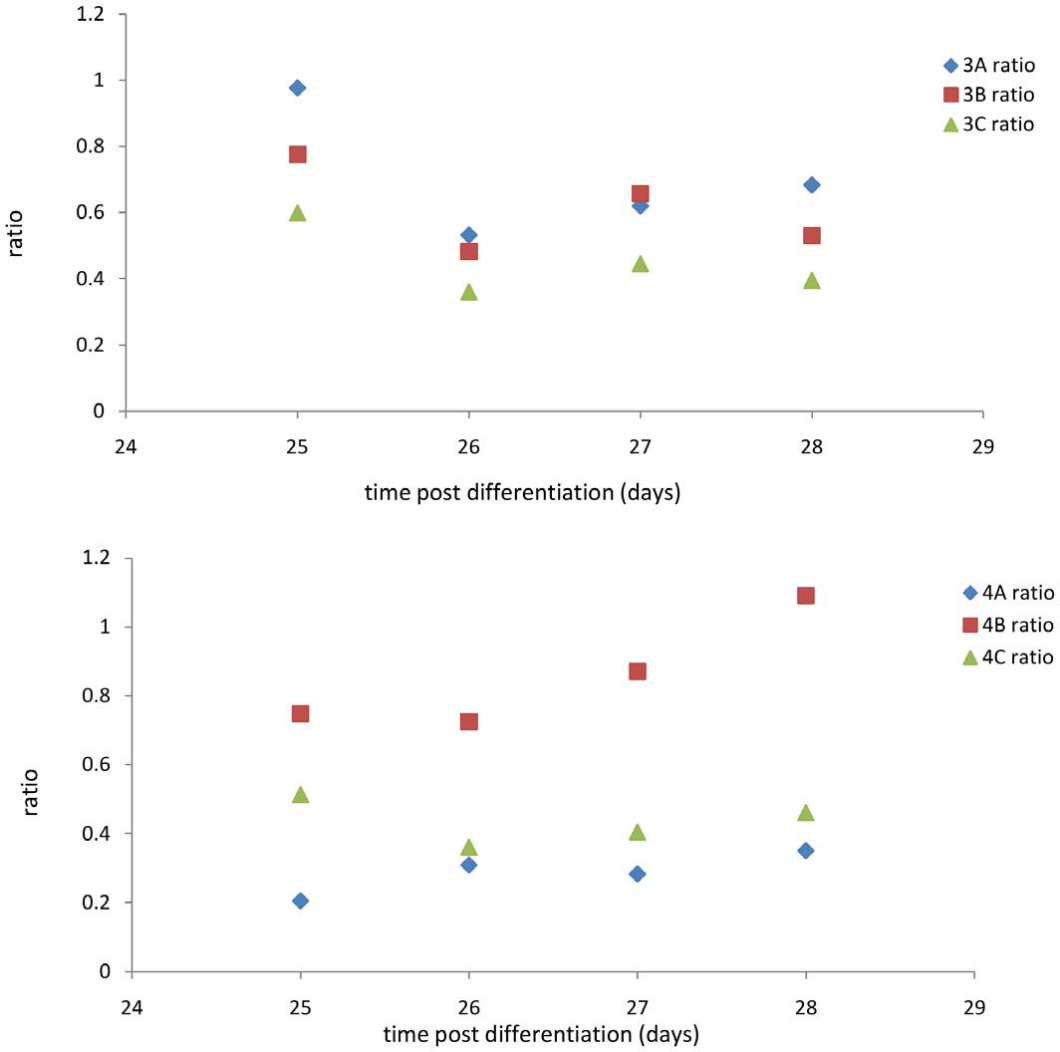
Differential mechanosensation response for Cohort 3 (upper graph) and Cohort 4 (lower graph). Each group (3A, 3B, 3C, 4A, 4B, 4C) was divided in half 20 days after the onset of differentiation, monolayers in each half either exposed to orbital shaking or not. The ratio of transepithelial sodium current of paired shaken and unshaken monolayers was used to quantify the long-time flow-sensing mechanosensation response. In all cases except 4B (discussed in the text), monolayers responded to the presence of flow by decreasing I_sc_ relative to monolayers in quiescent conditions. In particular, the differential response of Cohort 4 was attenuated over time, indicating that flow conditions post-differentiation have less of an effect than flow conditions present during differentiation.

Comparing the response of matched monolayers from cohorts 3 and 4 during the final stage of the flow protocol is shown in Figure 3.

Some cells were returned to the differentiation conditions (3A1, 3B1, 3C1; 4A2, 4B2, 4C2) while others were not. Ratioing the measured sodium current from paired monolayers returned to differentiation conditions to those that were not measured the degree to which monolayers maintained the ability to sense flow. This appears to be a long-lived feature of the cells, as shown by data presented in Figure 3. This data shows how much the sodium current changed in cohorts 3 and 4 during the final stage of the flow protocol: note that of the 6 groups only those subjected to constant flow conditions throughout the experiment (4B) lost the ability to distinguish between flow and no flow.

**Figure 4:**
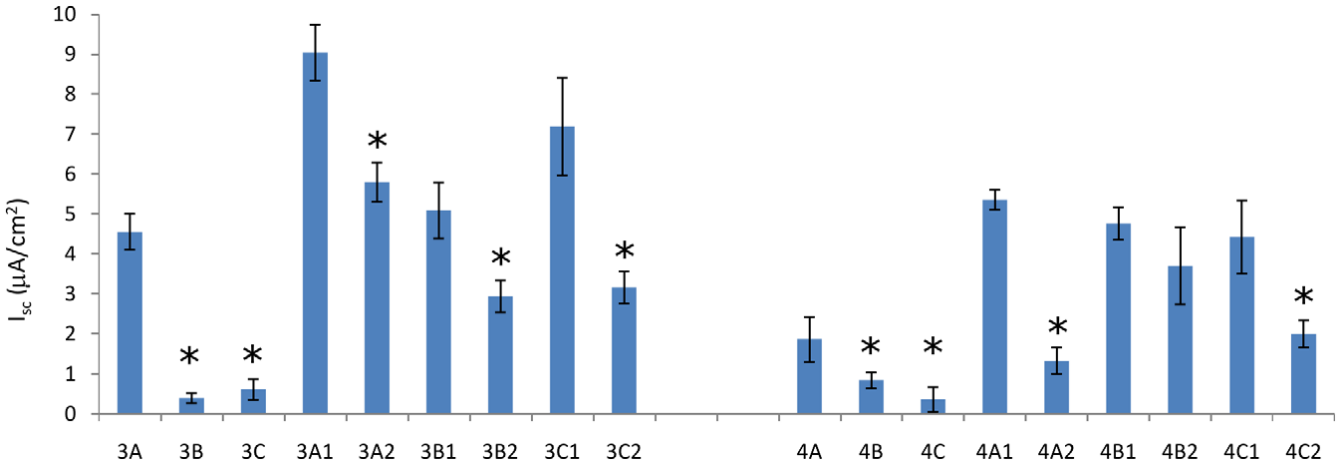
Time-averaged transepithelial sodium current compared across cohorts. This data compares the calculated short-circuit equivalent current I_sc_ within Cohorts 3 and 4 over time, illustrating the mechanosensation response as monolayers experience changes to the applied flow over time. With the exception of group 4B2, monolayers retained the ability to sense fluid flow as indicated by a significant decrease in I_sc_. Also of note is that cells exposed to flow during differentiation (Cohort 4) continue to have a decreased Isc relative to cells differentiated in the absence of flow (Cohort 3), and this difference persists over the entire experiment, even when monolayers from Cohort 4 are removed from flow for an extended period of time (e.g, comparing I_sc_ between groups 3A1 and 4A1).

Figure 4 presents the transepithelial Sodium current for the cohorts at different stages of the flow protocol. Each column is the average (over several days) of each set of monolayers. This data provides evidence regarding the chronic physiological response to changes in the flow conditions. Cohort 3, which differentiated in quiescent conditions, showed a strong (and single) response to the introduction of flow (3A vs. 3B and 3C) and later showed an increase in the sodium current, regardless of the presence or absence of flow. Cohort 4, differentiated in the presence of flow, overall had a lower amount of transepithelial sodium current regardless of the flow protocol. Of particular interest are the similar responses of samples 4A2 and 4C2: monolayers differentiated in the presence of flow, subjected to a change in flow, and then returned to differentiation conditions. These two data points indicate that the electrophysiological response of a cell to flow is not simply a proportional response (i.e. a higher rate of flow produces a lower sodium current), but rather that the cell responds to changes in the flow conditions in the same way, regardless if the flow increases (4A2) or decreases (4C2).

### Immunohistochemical results

The results provided here provide insight to the state of the cell only at the endpoint of the flow protocol; they provide information regarding the terminal state of the epithelial tissue. Note, although the various proteins were tagged with a wide spectrum of fluorescent secondaries as detailed in the methods section, for ease of comparison in images both cytoskeletal proteins (acetylated α-tubulin and actin) are rendered in green, both membrane proteins (Polycystin-2 and Somatostatin Receptor-3) are rendered in red, and the nucleus is rendered in blue.

#### Changing flow induces a proliferative response

**Figure 5:**
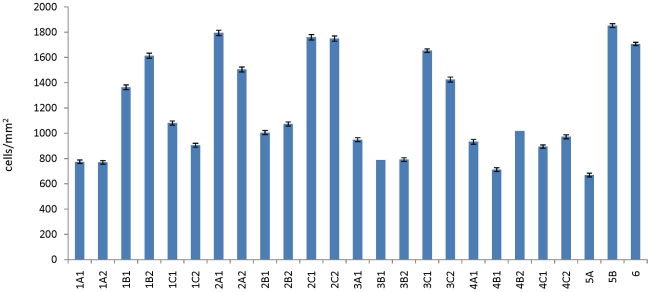
Measured cell density. At the conclusion of the experiment, monolayers were fixed and stained for immunohistochemical analysis. Monolayers were mounted with Vectashield containing DAPI, a nuclear stain. Monolayers were imaged at 10X magnification and the images thresholded for analysis. Three fields of view for each monolayer were obtained to improve statistics; each column is thus an average of at least 9 images (three images per monolayer, at least 3 monolayers per group). The data is not normally distributed, as verified by a quartile-quartile (Q-Q) plot; the data is instead two normal distributions, one with a mean of 870 +/− 130 cells/mm^2^ and the other with a mean of 1640 +/− 160 cells/mm^2^. The implication is that some monolayers proliferated for an additional complete cell cycle, specifically those monolayers experiencing a change in flow during the initial period of differentiation. Note that some groups are not presented due to a massive contamination event.

Figure 5 presents data from cell counting. Multiple images were acquired of each fixed monolayer, and the nuclei counted by a simple threshold operation. The cell density (cells/mm^2^) was not normally distributed, as verified by a Q-Q plot [38], but was strongly peaked about two values (870 +/− 130 cells/mm^2^ and 1640 +/− 160 cells/mm^2^). Because cell division halts when the cells become terminally differentiated, this data indicates that some cohorts take longer to differentiate than others- and further, the delay was sufficient for at least an additional cell cycle (8 hours).

This data provides suggestive evidence about the interplay between differentiation and fluid flow conditions. Cohorts 1 and 2 were exposed to flow either prior to differentiation or in the very early stages of differentiation. Cells hyperproliferated when flow conditions changed (1B, 2A, 2C), while cells did not proliferate when flow conditions did not change (1A, 2B). Cohort 1C did not show evidence of hyperproliferation, but the actin cystoskeleton of all the sub-groups of Cohort 1showed profound phenotypic differences with respect to all other cohorts (discussed below). Cohorts 3 and 4 were resistant to changes in flow conditions: the only group to hyperproliferate was subjected to maximal changes in flow conditions (no flow -> fast flow, group 3C). Of special interest are the results for cohorts 5B and 6- both showed evidence of hyperproliferation in spite of flow changes occurring well after (10 days) the onset of differentiation. The data presented here leads us to conclude that cells in a dynamic environment (i.e. the fluid flow rate is variable) can remain in a dedifferentiated pro-growth state, while cells exposed to a static environment- an environment where the fluid flow does not vary- stop proliferating and differentiate. These results should be considered together with other recent results [37] which demonstrate a clear link between ciliary function and cell division.

#### Actin cystoskeleton shows evidence of extensive remodeling in the presence of flow

**Figure 6:**
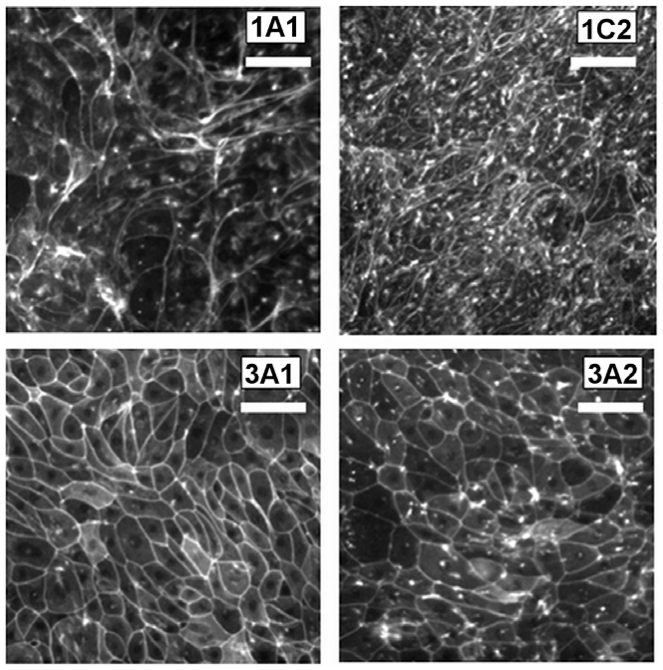
Immunostained actin cytoskeleton. The actin cytoskeleton was stained with phalloidin-Oregon Green and imaged at 10X to examine how the actin cytoskeleton may respond to orbital motion. Presented images are representative of all monolayers within a particular group (indicated on each image), and specific groups were chosen to illustrate representative cytoskeletal morphologies. In particular, monolayers subjected to high rates of motion for extended periods of time (1C2) presented a dense array of focal adhesions and stress fibers, while monolayers never subjected to flow (3A1) presented almost no focal adhesions and stress fibers. Monolayers initially subjected to flow and then removed (1A1) retained some focal adhesions and stress fibers, but in contrast to other groups presented a highly irregular and nonuniform morphology, specifically very large stress fiber bundles. Groups exposed to flow only at late stages, well past the onset of differentiation (3A2), developed numerous focal adhesions and only a few stress fibers. Although it is not clear if this mechanosensation response is correlated with the electrophysiological response, these images illustrate the dynamic interplay between the actin cystoskeleton and applied mechanical forces. Scale bar  = 10 µm.

Figure 6 shows the actin cystoskeleton from selected monolayers at low magnification (10X). Cohort 1C was exposed to fast flow immediately upon entering differentiation conditions, while Cohort 3 was only exposed to flow 10 days after the onset of differentiation. The images of cohort 1 shows extensive focal adhesions, irregular shapes, and stress fibers while images of all other cohorts lack the extensive focal adhesions and stress fibers.

Comparison of all monolayers either exposed to flow or not at the conclusion of the experiment show that in the presence of apical flow, increased numbers of focal adhesions are formed. This should be compared with the electrophysiological results during the same time period. Both cohorts 3 and 4 (which the exception of 4B) maintained the ability to sense flow, while group 4B lost the ability to sense flow. The visual appearance of the actin cystoskeleton provides evidence that focal adhesions (or integrin binding) may be correlated with the ciliary-mediated ENaC flow sensing response.

#### Cellular distributions of SSTR3 appear insensitive to flow while PC2 distributions depend on flow conditions

Based on considerable evidence presented in the literature [4], [7], [8], [16], [21], [25], [39], [40], I chose to measure the cellular distributions of Polycystin-2 and SSTR3 as a cellular readout. Both proteins are localized to the primary cilium and both participate in ciliary-mediated mechanosensation signaling pathways. Because protein localization is an acute response, the cellular localization of these proteins serves to measure the state of the cell just prior to fixation. That is, the data here does not necessarily correlate with the electrophysiological data presented above, except for the final time points.

**Figure 7:**
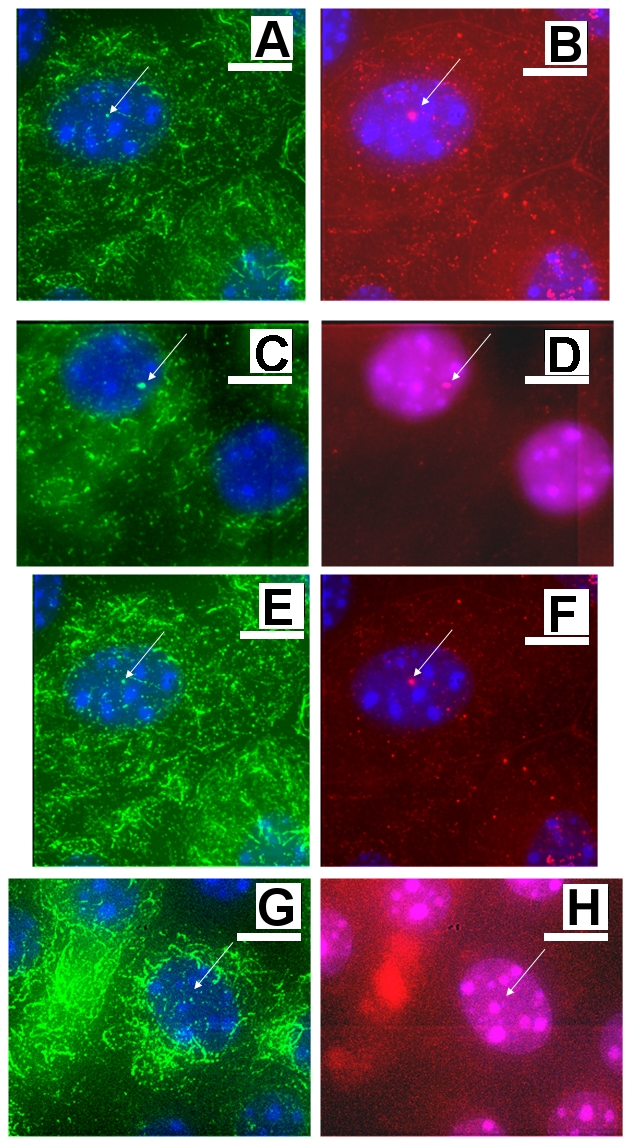
Localization of ciliary-associated mechanosensation proteins of interest. Fixed monolayers were stained for proteins known to associate with the ciliary-mediated flow response. Acetylated α-tubulin, displayed in green, is shown in (a), (c), (e), (g); SSTR3, displayed in red, is in (b) and (f); PC2, also imaged in red (see Methods section for a discussion), is in (d) and (h). DAPI stain is blue in all images. Furthermore, monolayers shown in (a)- (d) were not exposed to flow at the conclusion of the experiment while monolayers imaged in (e)-(h) were exposed to flow at the endpoint. The primary cilium is marked with arrows in all images. Comparison of images illustrates the ciliary-mediated flow response. Specifically, comparison of (b) and (f) demonstrates that SSTR3 remains in the primary cilium regardless of the flow conditions while (d) and (h) demonstrate that PC2 is trafficked away from the primary cilium in the presence of flow. These images provide evidence that the cell model can be used as a disease model for ciliary-mediated disease states, for example ADPKD. Scale bar  = 2 µm.

Figure 7 compares monolayers either subjected to flow or not, across selected cohorts. What is seen is that SSTR3 is localized to the primary cilium regardless of the flow conditions, while PC2 is localized to the primary cilium only when flow is present. In the absence of flow, no PC2 is detected in the primary cilium, in agreement with [41].

## Discussion

The magnitudes and durations fluid flow induced by orbital shaking approximately correspond to *in vivo* chronic stimulation within the mouse nephron (approximately 5 nl/min [42]). The readouts were chosen based on physiological relevance as well. Transepithelial Sodium current is a measure of the function of the cortical collecting duct. The collecting duct represents the final “fine-tuning” of salt and water homeostasis, and thus may play a role in chronic diseases that do not become pathological until late in life. Polycystin-2 is a ciliary-localized, membrane-bound Calcium channel that actively participates in the flow sensing pathway [8]. Actin may also participate in mechanosensation pathways [43]. Somatostatin Receptor-3 is a membrane-bound GPCR localized to the primary cilium only if proteins implicated in the human ciliary disorder Bardet–Biedl syndrome (BBS) are present [40].

### Electrophysiological results

Placing the results in the context of renal function, it is clear that there is significant interplay between applied flow conditions and salt resorption. The results show a clear response when the flow conditions change, and salt transport is decreased when flow is present. Furthermore, flow conditions present during differentiation affect the mechanosensation response later in time. *In vivo* conditions within the nephron are such that cellular differentiation proceeds while flow is present, thus the data presented here indicate that disease-relevant *in vitro* experiments should be conducted on tissue chronically subjected to flow during differentiation. While disease relevance has not been definitively established by this experiment, it is clear that flow conditions acting on undifferentiated tissue have long-lasting effects on differentiated renal epithelial tissue function. It is hypothesized that flow conditions within a tubule change during cystogenesis; this experiment begins to draw a mechanistic link between altered renal flow and salt transport. Specifically, salt transport increases in the absence of flow, and the chronic flow-sensing response is modulated by the flow conditions present at the onset of cellular differentiation. Combined with the hyperproliferation data, we begin to form a model: 1) changes in tubule flow drive increased cellular proliferation and 2) the cessation of flow causes increased salt transport. In this model, a tubule experiencing a dynamic flow environment will experience underregulated growth, leading to an overall decrease in flow rate, leading to increased salt resorption and hypertension.

It must be noted that under normal physiological conditions, tubule flow is highly dynamic due to changes in blood pressure over the course of a heartbeat. Importantly, the timescale of flow changes in this experiment are much longer and correlate to slow-acting chronic changes rather than the rapid changes associated with cardiac function.

Of note is the long-time behavior of cohort 4B: from the data presented in Figure 4, this cohort lost the ability to sense fluid flow. Examination of the α-tubulin immunostain showed that all the monolayers in this cohort lost their cilia. This is in agreement with previous results [36] which showed the cilium is required for fluid flow sensing. It is not known at this time why this particular cohort lost the cilia, but this particular cohort was subjected to flow from the onset of differentiation onwards andthe level of flow was not altered. Experiments should be performed to determine if this is a cause-effect relationship (chronic constant flow leading to loss of the cilium) or not, but is outside the scope of this paper. Taken together, the electrophysiological results indicate that 1) renal epithelial tissue requires apical fluid flow during differentiation to develop a graded response to flow, and 2) Sodium transport is modulated not only by the existence/absence of flow, but also changes in the magnitude of chronic flow.

### Immunostaining results

The actin images, taken with the protein localization images, show that actin remodeling is potentially independent from the ADPKD/BBS signaling pathway. Actin cytoskeleton remodeling is an acute response that occurs on a time scale of less than 10 days. Except for one case (Cohort 1C) which was exposed to high levels of flow at the onset of differentiation, the final state of the actin cytoskeleton is independent of the past history of flow conditions. It is not clear what the physiological relevance of this result is at this time, but the data provides evidence that actin remodeling may be an essential component of mechanosensation.

While the actin cystoskeleton shows extensive remodeling in response to physiological flow conditions, SSTR3 does not show any change in cellular distribution while PC2 does, in agreement with previous results [39], [41], [44]. The localization data is thus useful to establish the utility of this cell line as a potential disease model, as ciliary-associated proteins are correctly localized to the primary cilium and correctly trafficked, indicating normal physiological function.

### Induced Flow Conditions

**Figure 8:**
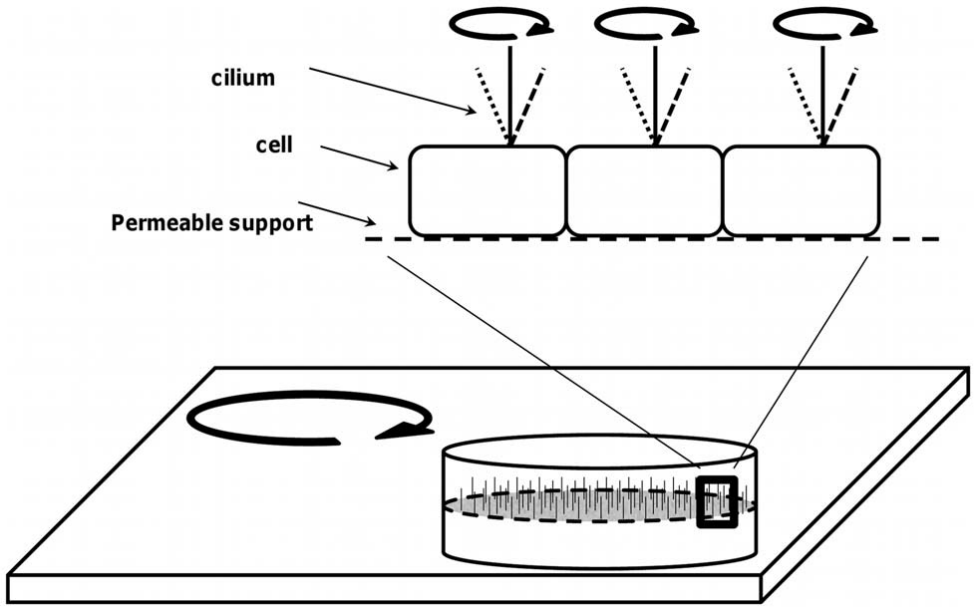
Orbital shaking geometry. Mouse Cortical Collecting Duct (mCCD) cells were grown to confluency on a suspended permeable membrane as described in the Methods section. Monolayers subjected to flow were placed atop an orbital shaker that chronically executed motion at a fixed (and known) frequency, also described within the Methods section. Orbital motion of the monolayers induced a gentle azimuthal fluid flow within the apical fluid layer, and this fluid flow is the primary mechanical stimulus to the primary cilium as discussed in the section “Induced Flow Conditions”.

The method used to induce apical flow is that same as presented in an earlier study [36]. To review, the experimental geometry for orbital shaking is shown in Figure 8. A confluent monolayer containing ciliated cells is placed atop an orbital shaker platform, which then executes orbital motion of constant throw (R) and user-controlled frequency (ω). At the low frequencies used in the study (≤5 Hz), the flow can be considered laminar.

Three types of mechanical forces due to the shaking are readily identified: 1) Shear stress on the apical membrane due to fluid movement relative to cells; 2) Drag force on the cilium due to fluid movement; and 3) Buoyancy induced by centripetal forces and density differences between the cilium and apical fluid, often referred to as “body force” because this force is distributed throughout the volume of the cilium. Calculations of the shear and drag forces require information on the fluid velocity profile between wall and cilia tips, in addition to fluid density, viscosity and ciliary geometry, while those for the buoyancy force require additional information about the density difference between cilium and fluid.

The fluid flow induced by gentle orbital shaking, particularly when the fluid layer above the cells is thin, constitutes a complex problem. To the best of my knowledge, neither an analytical nor a numerical solution of the flow field is available in the literature.

In our earlier study [36], we measured a time-averaged velocity (U) profile within the heights of the cilia as:
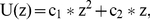
(1)with z indicating the height above the apical stationary surface. Coefficients ‘c_1_’ and ‘c_2_’ were determined by measurement and are unique to the orbital frequency. Once the velocity profile is known, the shear stress (τ) at the apical surface of the monolayer is defined by Equation 2:
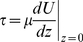
(2)where µ  =  fluid viscosity. The measured area for mCCD cells (from high resolution microscopy images, data not shown) is about 40 µm^2^. If this area is assumed as effective apical surface area, then the shear force on the entire cytoskeleton per cell is about 29 fN. However, because the cilium is required for flow sensing [36], the correct calculation is for the shear force at the cilium rather than the entire apical surface.

The drag force on a cilium can be calculated from fluid velocity transverse to the cilium assuming the cilium to be a rigid cylinder capped by a hemisphere and is given by Equation 3 [45]:
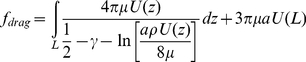
(3)where γ is Euler's constant (0.577…), ‘a’ the diameter of the cilium, ‘L’ the length of the cilium, and ρ  =  the density of the fluid. This expression can be numerically evaluated given a velocity profile U(z).

The buoyancy force comes about because of the relative motion between the monolayer frame and laboratory frame. Even if monolayer and fluid co-move (or if the cilium is free to pivot and is freely advected by the fluid), there will be a resultant force on the cilium due to centripetal acceleration. This force, averaged over time, is given by Eq. 4.
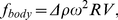
(4)Where V is the volume of the cilium.

Knowledge of the limits of applied forces is important for exploring the physiological roles of cilia in mechanosensation and ciliary pathophysiology in human disease, such polycystic kidney disease. The calculations were restricted to limit estimates because, as indicated above, no mathematical solution of the flow field is currently available for the type of geometry present in orbital shaking. We also need to emphasize that the elastic interaction between a cilium and the apical fluid is the subject of much research and a definitive picture has yet to emerge. I assume here, given the smallness of the applied forces, that the cilia do not move. If the cilia either flex or deflect in response to an applied force, then the applied force is even smaller than is calculated here, and so I consider the calculations to provide a maximal value.

Calculations were carried out for cilia with a length of 2.7 µm and a diameter of 0.2 µm. Following the calculations in [46], I present maximal estimates for the magnitudes of mechanical forces applied to a cilium in Table 1.

**Table 1:** Upper-bound magnitudes of flow-induced mechanical forces acting on a cilium.

Orbital frequency [Hz]	Shear force [fN]	Drag force [fN]	Bouyancy force [fN]
0.9	4.2*10^−2^	2.4	2.2*10^−5^
5	2.3*10^−1^	13.5	6.8*10^−4^

The primary conclusion is that the predominant force acting on the cilium is due to drag- the flow of fluid past the cilium.

## Methods

### Cell culture

Experiments were carried out with a mouse cell line derived following [47] from the cortical collecting duct (mCD 1296 (d)) of a heterozygous offspring of the Immortomouse (Charles River Laboratories, Wilmington, MA, USA). The Immortomouse carries as transgene a temperature-sensitive SV40 large T antigen under the control of an interferon-γ response element. Cells were maintained on collagen-coated Millicell-CM inserts (inner diameter 10 mm, permeable support area 0.6 cm^2^; Millipore Corp, Billerica, MA) to promote a polarized epithelial phenotype. Cells were grown to confluence at 33°C, 5% CO_2_ and then maintained at 39°C, 5% CO_2_ to enhance differentiation. The growth medium consisted of the following (final concentrations): Dulbecco's Modified Eagle Medium w/o glucose and Ham's F12 at a 1∶1 ratio, 5 mM glucose, 5 mg/ml transferrin, 5 mg/ml insulin, 10 µg/ml epithelial growth factor (EGF), 4 mg/ml dexamethasone, 15 mM 4-(2-hydroxyethyl)-1-piperazineethanesulfonic acid (HEPES), 0.06% NaHCO_3_, 2 mM L-glutamine, 10 ng/ml mouse interferon-γ, 50 µM ascorbic acid 2-phosphate, 20 nM selenium, 1 nM 3,3′,5-triiodo-L-thyronine (T3), 5%fetal bovine serum (FBS). For differentiation, FBS, insulin, and interferon-γ were omitted from the apical medium and insulin, EGF, and interferon-γ from the basal medium. The apical amount of medium was restricted to 100 µl per filter insert so that the apical fluid was thin enough to allow sufficient O_2_ to diffuse to the monolayer. All monolayers were routinely monitored for electrical resistance and transepithelial potential.

### Electrophysiology protocol

Transepithelial voltage and resistance measurements were performed using an Endohm chamber (World Precision Instruments, Sarasota, FL), connected to a MilliOhm (Millipore, Co.) voltohmmeter containing DMEM plus penicillin-streptomycin (10 mg/ml) and gentamicin (50 mg/ml). Cells were placed in the Endohm chamber for no more than 1 min, during which time the transepithelial voltage remained constant. The transepithelial voltage and resistance were converted to short-circuit current equivalent (I_sc_) assuming an ohmic relationship.

Sodium transport via ENaC is a well understood process that is described by the classical and, by now, text book Ussing model of sodium absorption: Sodium entry at the apical membrane is by electrodiffusion through the ENaC channel and exit at the basolateral membrane is by active transport via Na,K-ATPase in the basolateral plasma membrane. Due to the presence of potassium channels in the basolateral plasma membrane, Sodium transport by this process is completely electrogenic and can be quantified by measuring the short-circuit current (I_sc_) with identical electrolyte solutions on both sides of the epithelium. The current flow through ENaC is conveniently quantified by sensitivity of I_sc_ to 10 µM of apical amiloride, a well understood diuretic drug. Similarly, calculation of I_sc_ from transepithelial potential and resistance provides a quantitative measure of ENaC activity. More than 95% of I_sc_ was inhibited by 10 µM apical amiloride and thus I_sc_ is considered to be proportional to the activity of epithelial sodium channels (ENaC) in the apical plasma membrane.

### Immunocytochemistry

Fixation and immunocytochemistry were performed using standard techniques. The cells were briefly fixed in 4% paraformaldehyde, permeabilized for 10 min with a solution of 0.1% Triton-X and 0.5% saponin in a blocking buffer containing 5% donkey serum, 5% goat serum, 1% bovine serum albumin (BSA), and 5% fetal bovine serum (FBS). The monolayers were then stained with primary antibodies against proteins of interest (monoclonal mouse antibody against acetylated α-tubulin (Invitrogen, Carlsbad, CA), actin stained using phalloidin- Oregon green, polyclonal antibodies against Polycystin-2 anti-goat (H-280) and Somatostatin Receptor Type-3 (SSTR3) anti-rabbit (W-15), both supplied by Santa Cruz Biotechnology. The secondaries used to visualize the primary antibodies were Cy5 for acetylated α-tubulin (Invitrogen, Carlsbad, CA), DyLight 405 nm (Polycystin-2) and Texas Red (SSTR3), both supplied by Thermo Scientific. The stained filter was cut out of the culture insert and transferred to a microscope slide, monolayer side up. The filter was mounted in VectaShield (Vector Labs, Burlingame, CA) with DAPI. A # 1 ½ coverslip was placed on top of the monolayer- if needed, small spacers were used to prevent the coverslip from contacting the monolayer. The slides were then sealed with nail polish and stored for imaging at 4°C.

### Microscopy protocol

Image stacks were acquired with a Leica DM6000 B upright microscope using a 63X numerical aperture 1.47 Plan Apo lens with a Lumen 200 Pro source (Prior Scientific) and a Rolera-MGi Plus intensified camera (QImaging) under control of MicroManager, an open-source microscopy software package [48]. Stacks were deconvolved using an ImageJ plugin. Cell counting images were obtained with a 10X numerical aperture 0.3 fluar lens. Typical exposure times for individual frames were 200 ms and bleaching was minimal.

### Orbital motion protocol

Cells were subjected to orbital motion by placing monolayers on top of a shaker table (MTS 2/4, IKA corp., Wilmington, NC) placed within a standard laboratory incubator. Monolayers without shaking were maintained in the same incubator. Shaken monolayers were subjected to orbital motion at a fixed frequency (either 0.9 Hz (slow) or 5 Hz (fast)) determined by a stopwatch. Electrophysiological measurements or fixation for immunocytochemistry were carried out on cells immediately after removal from the orbital shaker.

**Figure 9:**
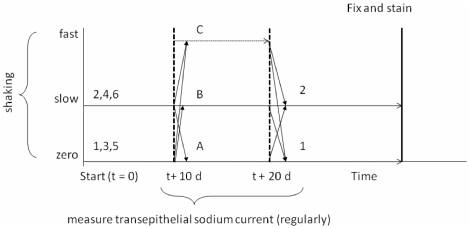
Flow protocol schematic. The purpose of the overall experiment was to probe the ciliary-mediated mechanosensation response to chronic fluid flow. Monolayers were also exposed to specific changes in flow rates to assess the dynamics of the mechanosensation response. Previous experiments have shown that the transepithelial sodium current is a valid readout that requires several days to reach steady-state conditions. Thus, monolayers were maintained in constant conditions for 10 day blocks of time. Finally, by choosing different start times relative to the onset of differentiation, the role of initial flow conditions as a modifier of future function could be assessed. In order to uniquely identify each group of monolayers for analysis, a three-digit alphanumeric code was assigned based on the particular pattern of applied flow conditions and start time relative to the onset of differentiation. Cohorts 1 and 2 started the protocol 72 hours prior to entering differentiation conditions, cohorts 3 and 4 began the protocol at the onset of differentiation conditions, and cohorts 5 and 6 began the flow protocol 10 days after the onset of differentiation conditions.

Broadly speaking, monolayers were exposed to a particular initial condition (flow or no flow) for 10 days, subjected to a second flow condition (no, slow, or fast flow) for 10 days, and finally were returned to either slow or no flow conditions until fixation (Figure 9). Each group was identified by a unique 3-character alphanumeric code presented in Table 2. The total number of confluent monolayers used was sufficient that at the conclusion of the experiment, each experimental condition was represented by at least 3 monolayers. Cohorts #1 and 2 began the protocol 24 hours after final plating, while the cells were still in the growth phase, 72 hours prior to confluency. Cohorts #3 and 4 began the protocol immediately upon confluency, and cohorts #5 and 6 began the protocol 10 days after confluency. The time period of 10 days was chosen based upon previous results [36] demonstrating that ENaC current stabilized after 10 days. Thus, the three paired cohorts represent different time slices of cellular differentiation and serve to probe the differentiation pathway.For example, cohort 3 began with 18 monolayers which were then split into three groups of 6 (3A, 3B, 3C) for the second phase and again split into six groups of 3 for the final phase (3A1, 3A2, 3B1, 3B2, 3C1, 3C2).

**Table 2:** Index of code assignments to the groups within cohorts.

Orbital Frequency [Hz]	Initial conditions	Intermediate conditions	Terminal conditions
5	n/a	C	n/a
0.9	2,4,6	B	2
0 (static)	1,3,5	A	1

One consequence of this protocol is that each initial cohort had some subgroups that followed the same experimental protocol as monolayers in other cohorts: for example, monolayers 1A1 were never exposed to orbital motion, similar to monolayers in groups 3A1 and 5A1. Similarly, monolayers 2B2, 4B2, 5B2 were continuously shaken at the same frequency throughout (although those groups spent varying amounts of time in initial quiescent conditions). Thus, the protocol established internal positive and negative controls.

### Viscosity measurement protocol

The dynamic viscosity of apical media was measured with a Cannon-Fenske Routine Viscometer (Induchem Lab Glass, Inc., Roselle, NJ) with the apparatus and media equilibrated to 37°C. The density of the media was measured by weighing a controlled volume (100 µl Dummond Wiretrol disposable micropipette, Dummond Scientific Company, Broomall, PA). In both cases, the apparatus was calibrated by measurements on double distilled water to ensure accuracy.

### Statistical Analysis

Data is presented with +/− 1 standard deviation error bars. Statistical analysis was done by Student's *t*-test, two-tailed heteroscedastic, as appropriate. Values of p<0.01 are considered significant. Groups with fewer than 3 monolayers were omitted from presented results.

### Conclusion and Future work

The primary finding of interest here is that cells differentiated in the presence of flow behave very differently from cells differentiated in the absence of flow. Additionally,our cell line displays highly physiologically relevant behavior in the presence of fluid flow: the transepithelial sodium current is modulated, the actin cystoskeleton remodels, and certain membrane-bound proteins localize to the primary cilium. Taken together, our results begin to show a pathophysiological mechanism by which tubular cells hyperproliferate and form fluid-filled cysts: salt transport is decreased, leading to an accumulation of ultrafiltrate on the apical side of the epithelium. The presented data also supports the use of this cell line as a disease model. Future work will involve subjecting cells to a more defined flow by culturing the cells in a flow chamber rather than subjecting the cells to orbital motion. Additionally, further work determining the protein localization of Polycystin-1 (which cleaves in the presence of flow), STAT6 (which also traffics between the nucleus and cilium in the presence of flow) will be performed.

## References

[1] Rieder CL, Faruki S, Khodjakov A (2001). The centrosome in vertebrates: more than a microtubule-organizing center.. Trends Cell Biol.

[2] Satir P (1961). Cilia.. Sci Am.

[3] Alberts B (2008). Molecular biology of the cell, 5th edition.. Extended version.

[4] Handel M, Schulz S, Stanarius A, Schreff M, Erdtmann-Vourliotis M (1999). Selective targeting of somatostatin receptor 3 to neuronal cilia.. Neuroscience.

[5] Calvet JP (2003). New insights into ciliary function: kidney cysts and photoreceptors.. Proc Natl Acad Sci U S A.

[6] Lin F, Hiesberger T, Cordes K, Sinclair AM, Goldstein LS (2003). Kidney-specific inactivation of the KIF3A subunit of kinesin-II inhibits renal ciliogenesis and produces polycystic kidney disease.. Proc Natl Acad Sci U S A.

[7] Luo Y, Vassilev PM, Li X, Kawanabe Y, Zhou J (2003). Native polycystin 2 functions as a plasma membrane Ca2+-permeable cation channel in renal epithelia.. Mol Cell Biol.

[8] Nauli SM, Alenghat FJ, Luo Y, Williams E, Vassilev P (2003). Polycystins 1 and 2 mediate mechanosensation in the primary cilium of kidney cells.. Nat Genet.

[9] Marszalek JR, Ruiz-Lozano P, Roberts E, Chien KR, Goldstein LS (1999). Situs inversus and embryonic ciliary morphogenesis defects in mouse mutants lacking the KIF3A subunit of kinesin-II.. Proc Natl Acad Sci U S A.

[10] Murcia NS, Richards WG, Yoder BK, Mucenski ML, Dunlap JR (2000). The Oak Ridge Polycystic Kidney (orpk) disease gene is required for left-right axis determination.. Development.

[11] Whitfield JF (2008). The solitary (primary) cilium-A mechanosensory toggle switch in bone and cartilage cells.. Cell Signal.

[12] Fischer E, Pontoglio M (2009). Planar cell polarity and cilia.. Semin Cell Dev Biol.

[13] Berbari NF, O'Connor AK, Haycraft CJ, Yoder BK (2009). The primary cilium as a complex signaling center.. Curr Biol.

[14] Pan J, Wang Q, Snell WJ (2005). Cilium-generated signaling and cilia-related disorders.. Lab Invest.

[15] Pazour GJ (2004). Intraflagellar transport and cilia-dependent renal disease: the ciliary hypothesis of polycystic kidney disease.. J Am Soc Nephrol.

[16] Praetorius HA, Frokiaer J, Nielsen S, Spring KR (2003). Bending the primary cilium opens Ca2+-sensitive intermediate-conductance K+ channels in MDCK cells.. J Membr Biol.

[17] Praetorius HA, Spring KR (2001). Bending the MDCK cell primary cilium increases intracellular calcium.. J Membr Biol.

[18] Praetorius HA, Spring KR (2003). Removal of the MDCK cell primary cilium abolishes flow sensing.. J Membr Biol.

[19] Taulman PD, Haycraft CJ, Balkovetz DF, Yoder BK (2001). Polaris, a protein involved in left-right axis patterning, localizes to basal bodies and cilia.. Mol Biol Cell.

[20] Hou X, Mrug M, Yoder BK, Lefkowitz EJ, Kremmidiotis G (2002). Cystin, a novel cilia-associated protein, is disrupted in the cpk mouse model of polycystic kidney disease.. J Clin Invest.

[21] Yoder BK, Hou X, Guay-Woodford LM (2002). The polycystic kidney disease proteins, polycystin-1, polycystin-2, polaris, and cystin, are co-localized in renal cilia.. J Am Soc Nephrol.

[22] Brown NE, Murcia NS (2003). Delayed cystogenesis and increased ciliogenesis associated with the re-expression of polaris in Tg737 mutant mice.. Kidney Int.

[23] Siroky BJ, Ferguson WB, Fuson AL, Xie Y, Fintha A (2006). Loss of primary cilia results in deregulated and unabated apical calcium entry in ARPKD collecting duct cells.. Am J Physiol Renal Physiol.

[24] Cano DA, Murcia NS, Pazour GJ, Hebrok M (2004). Orpk mouse model of polycystic kidney disease reveals essential role of primary cilia in pancreatic tissue organization.. Development.

[25] Liu W, Murcia NS, Duan Y, Weinbaum S, Yoder BK (2005). Mechanoregulation of intracellular Ca2+ concentration is attenuated in collecting duct of monocilium-impaired orpk mice.. Am J Physiol Renal Physiol.

[26] Pei Y (2001). A "two-hit" model of cystogenesis in autosomal dominant polycystic kidney disease?. Trends Mol Med.

[27] Low SH, Vasanth S, Larson CH, Mukherjee S, Sharma N (2006). Polycystin-1, STAT6, and P100 function in a pathway that transduces ciliary mechanosensation and is activated in polycystic kidney disease.. Dev Cell.

[28] Andrade YN, Fernandes J, Vazquez E, Fernandez-Fernandez JM, Arniges M (2005). TRPV4 channel is involved in the coupling of fluid viscosity changes to epithelial ciliary activity.. J Cell Biol.

[29] Satlin LM, Sheng S, Woda CB, Kleyman TR (2001). Epithelial Na(+) channels are regulated by flow.. Am J Physiol Renal Physiol.

[30] Becker JU, Saez AO, Zerres K, Witzke O, Hoyer PF (2010). The mTOR pathway is activated in human autosomal-recessive polycystic kidney disease.. Kidney Blood Press Res.

[31] Shillingford JM, Murcia NS, Larson CH, Low SH, Hedgepeth R (2006). The mTOR pathway is regulated by polycystin-1, and its inhibition reverses renal cystogenesis in polycystic kidney disease.. Proc Natl Acad Sci U S A.

[32] Weimbs T (2006). Regulation of mTOR by polycystin-1: is polycystic kidney disease a case of futile repair?. Cell Cycle.

[33] Weimbs T (2007). Polycystic kidney disease and renal injury repair: common pathways, fluid flow, and the function of polycystin-1.. Am J Physiol Renal Physiol.

[34] Bonventre JV (2003). Dedifferentiation and proliferation of surviving epithelial cells in acute renal failure.. J Am Soc Nephrol.

[35] Guo JK, Cantley LG (2010). Cellular maintenance and repair of the kidney.. Annu Rev Physiol.

[36] Resnick A, Hopfer U (2007). Force-response considerations in ciliary mechanosensation.. Biophys J.

[37] AbouAlaiwi WA, Ratnam S, Booth RL, Shah JV, Nauli SM (2011). Endothelial cells from humans and mice with polycystic kidney disease are characterized by polyploidy and chromosome segregation defects through survivin down-regulation.. Hum Mol Genet.

[38] Blom G (1958). Statistical estimates and transformed beta-variables..

[39] Wang S, Zhang J, Nauli SM, Li X, Starremans PG (2007). Fibrocystin/polyductin, found in the same protein complex with polycystin-2, regulates calcium responses in kidney epithelia.. Mol Cell Biol.

[40] Berbari NF, Lewis JS, Bishop GA, Askwith CC, Mykytyn K (2008). Bardet-Biedl syndrome proteins are required for the localization of G protein-coupled receptors to primary cilia.. Proc Natl Acad Sci U S A.

[41] Singla V, Reiter JF (2006). The primary cilium as the cell's antenna: signaling at a sensory organelle.. Science.

[42] Meneton P, Ichikawa I, Inagami T, Schnermann J (2000). Renal physiology of the mouse.. Am J Physiol Renal Physiol.

[43] Duan Y, Gotoh N, Yan Q, Du Z, Weinstein AM (2008). Shear-induced reorganization of renal proximal tubule cell actin cytoskeleton and apical junctional complexes.. Proc Natl Acad Sci U S A.

[44] Iwanaga T, Miki T, Takahashi-Iwanaga H (2011). Restricted expression of somatostatin receptor 3 to primary cilia in the pancreatic islets and adenohypophysis of mice.. Biomed Res.

[45] Lamb H (1945). Hydrodynamics..

[46] Resnick A, Hopfer U (2008). Mechanical stimulation of primary cilia.. Front Biosci.

[47] Kolb RJ, Woost PG, Hopfer U (2004). Membrane trafficking of angiotensin receptor type-1 and mechanochemical signal transduction in proximal tubule cells.. Hypertension.

[48] Edelstein A, Amodaj N, Hoover K, Vale R, Stuurman N (2010). Computer control of microscopes using microManager.. Curr Protoc Mol Biol Chapter.

